# Unscheduled return visits to a Dutch inner-city emergency department

**DOI:** 10.1186/s12245-014-0023-6

**Published:** 2014-07-05

**Authors:** M Christien van der Linden, Robert Lindeboom, Rob de Haan, Naomi van der Linden, Ernie RJT de Deckere, Cees Lucas, Steven J Rhemrev, J Carel Goslings

**Affiliations:** 1 Accident and Emergency Department, Medical Centre Haaglanden, The Hague 2501 CK, The Netherlands; 2 Division of Clinical Methods and Public Health, Master Evidence Based Practice, Academic Medical Centre, University of Amsterdam, Amsterdam 1100 DD, The Netherlands; 3 Clinical Research Unit, Academic Medical Centre, University of Amsterdam, J1b-118, Amsterdam 1100 DD, The Netherlands; 4 Institute for Medical Technology Assessment, Erasmus University Rotterdam, Rotterdam 3000 DR, The Netherlands; 5 Department of Surgery, Trauma Unit, Academic Medical Centre, University of Amsterdam, Amsterdam 1100 DD, The Netherlands

**Keywords:** Emergency service, Hospital, Emergency department, Unscheduled return visits

## Abstract

**Background:**

Unscheduled return visits to the emergency department (ED) may reflect shortcomings in care. This study characterized ED return visits with respect to incidence, risk factors, reasons and post-ED disposition. We hypothesized that risk factors for unscheduled return and reasons for returning would differ from previous studies, due to differences in health care systems.

**Methods:**

All unscheduled return visits occurring within 1 week and related to the initial ED visit were selected. Multivariable logistic regression was conducted to determine independent factors associated with unscheduled return, using patient information available at the initial visit. Reasons for returning unscheduled were categorized into illness-, patient- or physician-related. Post-ED disposition was compared between patients with unscheduled return visits and the patients who did not return.

**Results:**

Five percent (*n* = 2,492) of total ED visits (*n* = 49,341) were unscheduled return visits. Patients with an urgent triage level, patients presenting during the night shift, with a wound or local infection, abdominal pain or urinary problems were more likely to return unscheduled. Reasons to revisit unscheduled were mostly illness-related (49%) or patient-related (41%). Admission rates for returning patients (16%) were the same as for the patients who did not return (17%).

**Conclusions:**

Apart from abdominal complaints, risk factors for unscheduled return differ from previous studies. Short-term follow-up at the outpatient clinic or general practitioner for patients with urgent triage levels and suffering from wounds or local infections, abdominal pain or urinary problem might prevent unscheduled return.

## Background

Unscheduled return visits to the emergency department (ED) are visits of patients who were seen in the ED and then return for an unscheduled visit for the same complaint. Unscheduled return visits may reflect a failure of the patients' treatment or discharge plan [[1]]. Different numbers of unscheduled return visits have been reported, ranging from 2% to 5% of the patients returning to the ED within 2 to 8 days after their initial visit [[2]–[9]]. The reasons for unscheduled return are frequently grouped into illness-related factors (such as disease progression), patient-related factors (such as patients who left against medical advice during their initial visit) and physician-related factors (such as medical errors) [[3],[6],[7]]. Unscheduled return visits are more common in patients who lack access to primary care [[10]] and in patients with no health insurance [[11]]. Unscheduled return is associated with frequent ED use [[12]] and a greater risk of adverse events and mortality [[13]].

In order to reduce unscheduled return visits, researchers have focused on risk factors that could help identify patients at risk for unscheduled return [[11],[14]–[19]]. Most of these studies have been performed in Canada and the USA and reported acute triage category [[14],[16]], arrival in the evening [[14]] and a respiratory diagnosis [[19]] as risk factors for paediatric unscheduled return. A digestive diagnosis was reported as risk factor for unscheduled return in patients 65 years of age or older [[17],[18]]. Having no insurance, a low triage category and suffering from dermatologic conditions [[11]] were risk factors for unscheduled return in a mixed (adults and children) population.

In the Netherlands, the incidence of unscheduled ED return is unknown. We expect however that the incidence is lower than described in previous studies. Because all Dutch citizens have a general practitioner (GP) and GP services are available 24/7, patients should present at their GP instead of at the ED when they have ongoing complaints. We also hypothesize that the type of risk factors associated with unscheduled return differs from other studies, given the difference in health care systems. Health insurance is compulsory for all Dutch citizens, and health insurers are obliged to accept anyone who applies for standard health insurance.

The objectives of this study were to determine the incidence of unscheduled ED return visits, to identify the risk factors for these return visits, to assess the reasons for unscheduled return and to describe the post-ED disposition of patients at their return visit.

## Methods

The study was conducted between 1 October 2009 and 30 September 2010 at the ED of Medical Centre Haaglanden, the Hague, the Netherlands, an urban, 380-bed trauma centre. The annual volume in the ED is approximately 52,000 visits, with a 17% admission rate.

The following are the methods of measurement used for each objective of the study:

1. To determine the incidence of ED return visits, we performed a database search of the patients' records. Emergency department return visits were included if they took place within 1 week of the initial visit and concerned the same complaint or its direct consequences. Scheduled return visits (visits of patients who were told to come back to the ED) were excluded.

2. To identify factors associated with unscheduled return, we manually reviewed all individual patient charts and compared patients with unscheduled return visits with patients who did not return. We examined factors (available at the initial visit) that were associated with unscheduled return in previous research, including age [[14],[20]], sex [[17],[20]], lacking health insurance [[11]], lacking a GP [[10]], triage level [[11],[14],[16],[20]], arrival time [[14],[21]], length of stay (LOS) [[22]] and medical complaints [[11],[15],[18]]. Medical complaints for which a patient visited the ED were retrieved by the triage flow charts recorded by the triage nurse.

3. Reasons for returning unscheduled were categorized into illness-related, patient-related or physician-related (Table 1), based on examples from previous studies [[6],[9],[23]]. Categorization was independently done by two researchers (MCL and NL). In case of no agreement, the case was reviewed by a third researcher (ERJTD) and assigned to the category on which two of the three researchers agreed.

**Table 1:** Reasons for unscheduled return and definitions

	**Definition**
Physician-related return	
No painkillers prescribed	The disease or injury warranted pain medication but no prescription was given. The patient returned primarily because of continued pain
Treatment error	The physician made the right diagnosis during the initial visit, but made an error in treatment
Misdiagnosis	Medical record review reveals a diagnosis or problem missed by the physician who saw the patient on the initial visit
Patient-related return	
Left against medical advice	The patient was seen by a physician and left the ED against medical advice
Non-compliance	There is evidence in the medical records that the patient did not follow instructions
Psychiatric disorder and/or substance abuse	The patient has a psychiatric disorder and/or uses drugs or alcohol, which causes him/her to repeatedly visit the ED for the same or similar problems. Mentally, the patient is in a chronic stable state
Left without being seen	The patient was registered in the ED but left before being seen by a physician
Patient was instructed to visit own GP	The patient was instructed to return to the GP for re-evaluation but did not go
Worrying about health	The patient's anxiety caused him/her to return to the ED for the same or similar problem. No ancillary diagnostics were performed and medical management consisted of reassurance only
Illness-related return	
Recurrent disease process	The patient has a disease that tends to have recurrent exacerbations (i.e. asthma, sickle cell disease). The patient was treated appropriately during the initial ED visit, with resolution of symptoms, but later returned with a second exacerbation of the disease
Complication	The patient was treated appropriately during the initial ED visit but returned to the ED because of a complication of the disease or unpredictable side effect of treatment (e.g. allergic drug reaction)
Progression of disease	The medical records reveal that the patient was treated appropriately at the initial visit and that admission was not indicated. Appropriate follow-up was arranged, but the patient's disease or problem got worse, and he/she returned to the ED as instructed
Ancillary diagnostics performed, no change in diagnosis	The patient presented with the same or similar problem, ancillary diagnostics were performed but there was no change in the initial diagnosis or treatment

4. Post-ED dispositions were the discharge codes after the patients' treatment at the ED, comprising discharge, discharge against medical advice or left without being seen, hospital admission to a regular ward or admission to a special care unit (intensive care, coronary care or stroke unit).

All variables were obtained from the hospital electronic database and the medical records. The analyzed patient dataset contained no individual identifiers, maintaining anonymity of subjects. This study was approved by the institutional review board.

### Analysis

Patient and clinical characteristics were summarized using simple descriptive statistics. The *χ*^2^ test and unadjusted odds ratios (ORs) were used to assess the univariate association between age, sex, lacking health insurance, lacking a GP, triage level, arrival time, LOS and medical complaints on the one side and unscheduled return within 1 week on the other side. Additionally, all variables that were univariately associated with unscheduled return at ≤0.05 were entered into a multivariate logistic regression model. We also did the analysis with a <72-h unscheduled return. Effect sizes were expressed in adjusted ORs. The calibration and overall discriminative ability of the model was assessed with the Hosmer-Lemeshow test and the area under the receiver operating curve (AUC ROC) analysis, respectively [[24]]. In all analyses, statistical uncertainty was expressed in a 95% confidence interval (CI). Statistical analyses were performed in PASW (Predictive Analytics Software, version 18, Chicago, IL, USA).

## Results

### Return rate

**Figure 1:**
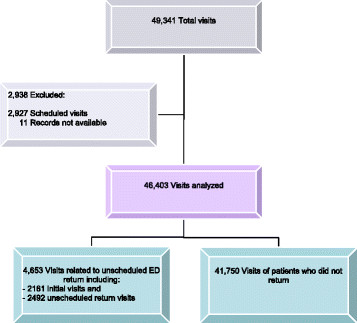
Flow chart: number of visits and repeat visits.

During the study year, a total of 49,341 ED visits were recorded, of which 4,653 visits were related to unscheduled return (Figure 1). In total, 2,161 patients returned unscheduled to the ED within a week of their initial registration. Since some of them returned more than once, there were 2,492 associated unscheduled return visits, comprising 5.1% of the total ED visits (2,492/49,341).

During the first 72 h after the initial visit, 1,279 patients made 1,330 return visits out of 49,341 total ED visits for an overall 72-h return rate of 2.7%.

### Factors associated with unscheduled return

Table 2 shows the univariate and multivariate associations between patient/clinical characteristics available at the initial visit and unscheduled ED return within 1 week. Logistic regression showed that the following factors had an independent impact on within-week unscheduled return: ‘urgent triage level’, ‘arrival during the night’, ‘LOS >1 h’ and the medical complaints ‘wound or local infections’, ‘abdominal pain’ and ‘urinary problems’ at the initial visit. Patients suffering from ‘chest pain’, ‘feeling unwell’ and children triaged with the category ‘sick baby’ were less likely to return unscheduled. The goodness of fit of the logistic model was moderate (*P* = 0.75), whereas the AUC demonstrated a weak discriminative ability (0.57; 95% CI 0.56 to 0.59).

**Table 2:** Characteristics associated with unscheduled ED return: univariate and multivariate analysis

	**Patients who did not return**	**Patients with unscheduled return visits**	**Unadjusted odds ratio**^**a,b**^**(95% CI),** ***P*** **value**	**Adjusted odds ratio**^**a,b,c**^**(95% CI),** ***P*** **value**
	**(*n*** **= 41,750)**	**(*n*** **= 2,161)**		
Age [mean (standard deviation)]	38.2 (22.3)	39.3 (20.7)	1.00 (1.00, 1.00), 0.03	-^e^
Sex, male [*n* (%)]	21,572 (51.7)	1,155 (53.4)	1.07 (0.99, 1.17), 0.11	-
Lacking health insurance [*n* (%)]	1,714 (4.1)	97 (4.5)	1.01 (0.89, 1.35), 0.38	-
Lacking a GP [*n* (%)]	3,255 (7.8)	155 (7.2)	0.91 (0.77, 1.08), 0.29	-
Triage level [*n* (%)]				
Levels 1 and 2	6,482 (16.1)	298 (14.2)	1.00 (0.88, 1.15), 0.96	1.13 (0.97, 1.32), 0.12
Level 3	13,324 (33.1)	859 (41.0)	1.41 (1.28, 1.55), <0.01	1.40 (1.26, 1.55), <0.01
Levels 4 and 5 (reference category)	20,428 (50.8)	936 (44.7)	1	1
No triage^c^ [*n* (%)]	1,516 (3.6)	68 (3.1)	0.86 (0.67, 1.10), 0.24	-
Arrival time [*n* (%)]				
Day, 7.30 a.m. to 3.29 p.m. (reference category)	17,844 (42.7)	882 (40.8)	1	1
Evening, 3.30 p.m. to 10.59 p.m.	18,193 (43.6)	925 (42.8)	1.03 (0.94, 1.13), 0.56	1.03 (0.94, 1.14), 0.54
Night, 11.00 p.m. to 7.29 a.m.	5,713 (13.7)	354 (16.4)	1.25 (1.10, 1.42), <0.01	1.24 (1.09, 1.41), <0.01
Length of stay [*n* (%)]				
<1 h (reference category)	9,918 (23.8)	435 (20.1)	1	-
1 to 2 h	11,966 (28.7)	648 (30.0)	1.24 (1.09, 1.40), <0.01	1.25 (1.09, 1.42), 0.00
2 to 3 h	8,804 (21.1)	452 (20.9)	1.17 (1.02, 1.34), 0.02	1.16 (1.00, 1.34), 0.05
3 to 4 h	5,001 (12.0)	283 (13.1)	1.29 (1.11, 1.50), <0.01	1.26 (1.06, 1.48), 0.01
>4 h	6,061 (14.5)	343 (15.9)	1.29 (1.12, 1.49), <0.01	1.24 (1.05, 1.45), 0.01
Medical complaint [*n* (%)]				
Extremity-related complaints	9,789 (23.4)	498 (23.0)	0.98 (0.88, 1.08), 0.67	-
Wounds and local infections	4,726 (11.3)	281 (13.0)	1.17 (1.03, 1.33), 0.02	1.34 (1.17, 1.54), <0.01
Other^d^	4,480 (10.7)	225 (10.4)	0.97 (0.84, 1.11), 0.64	-
Abdominal pain	3,597 (8.6)	269 (12.4)	1.51 (1.32, 1.72), <0.01	1.38 (1.20, 1.59), <0.01
Chest pain	3,547 (8.5)	146 (6.8)	0.78 (0.66, 0.93), <0.01	0.78 (0.64, 0.94), 0.01
Feeling unwell	3,124 (7.5)	131 (6.1)	0.80 (0.67, 0.96), <0.01	0.75 (0.62, 0.91), 0.00
Eye/ear/nose problems and sore throat	2,317 (5.5)	107 (5.0)	0.89 (0.73, 1.08), 0.24	-
Shortness of breath	2,085 (5.0)	99 (4.6)	0.91 (0.74, 1.12), 0.39	-
Headache and head injury	1,943 (4.5)	98 (4.7)	0.97 (0.79, 1.20), 0.80	-
Back pain	826 (2.0)	37 (1.7)	0.86 (0.62, 1.20), 0.39	-
Trauma, severe	771 (1.8)	32 (1.5)	0.80 (0.56, 1.14), 0.22	-
Psychiatric problem/substance abuse	685 (1.6)	44 (2.0)	1.25 (0.92, 1.70), 0.16	-
Rashes	660 (1.6)	32 (1.5)	0.94 (0.66, 1.34), 0.72	-
Urinary problems	641 (1.5)	59 (2.7)	1.80 (1.37, 2.36), <0.01	1.72 (1.31, 2.26), <0.01
Sick baby	524 (1.3)	12 (0.6)	0.44 (0.25, 0.78), <0.01	0.47 (0.27, 0.84), 0.01
No medical complaint registered	2,035 (4.9)	91 (4.2)	0.86 (0.69, 1.06), 0.16	-

^a^Categorical variables (triage level, arrival time and categorized LOS) were entered as ‘dummy’ variables. ^b^*χ*^2^ test, OR > 1 indicates an increased risk of unscheduled return. ^c^Adjusted for included variables (age, triage level, arrival time, LOS, medical complaint) by logistic regression model, based on 42,327 observations (40,234 visits of patients who did not return and 2,093 unscheduled return visits) due to missing values on triage level (*n* = 1,584). ^d^Medical complaints occurring less than 500 times per year (including allergy, dental problems, diabetes, exposure to chemicals, fits, neck pain, pregnancy, sexually acquired infections, testicular pain and vaginal bleeding) were categorized as ‘Other’. ^e^Not in multivariable model.

Sub-analysis of 72-h return showed that associated factors were the same as for within-week return (data not shown).

### Reasons for unscheduled return

**Figure 2:**
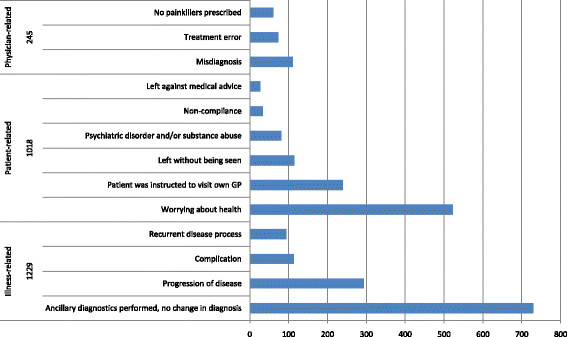
**Reasons for unscheduled return (*n*** **= 2,492 visits).**

The most common reasons for unscheduled return were illness-related (*n* = 1,229; 49%), followed by patient-related (*n* = 1,018; 41%) and physician-related reasons (*n* = 245; 10%) (Figure 2).

Within the 1,229 illness-related unscheduled return visits, ‘patients in whom ancillary diagnostics was performed while their diagnosis remained unchanged’ was the largest subgroup (*n* = 729; 59%). Within the 1,018 patient-related return visits, patients ‘worrying about health’ represented the most frequently occurring reason for return (523 visits, 51%). Within the physician-related return visits, 111 patients (45%) had ‘wrong or delayed diagnoses’ during their initial visit, which resulted in their return. In 73 physician-related return visits (30%), a ‘treatment error’ was made during the initial visit, such as patients returning with ongoing complaints because a foreign body in a wound was only removed partially. Sixty-one visits (25%) were caused by a ‘lack of a prescription of painkillers’ at the patients' initial visit.

### Post-ED disposition

No differences in post-ED disposition were found between patients with unscheduled return visits and patients who did not return (Table 3). Sixteen percent of the unscheduled return visits resulted in admission, versus 17% of the visits of patients who did not return.

**Table 3:** Post-ED disposition

	**Post-ED disposition after a visit of a patient who did not return (*n*** **= 41,750)**	**Post-ED disposition after an unscheduled return visit (*n*** **= 2,492)**	***P*** **value**^**a**^
Discharge [*n* (%)]	33,770 (80.9)	2,037 (81.7)	0.29
Hospital admission, regular ward [*n* (%)]	7,145 (17.1)	401 (16.1)	0.19
Hospital admission, special care^b^ [*n* (%)]	76 (0.2)	2 (0.1)	0.24
Discharge against medical advice or LWBS^c^ [*n* (%)]	727 (1.7)	52 (2.1)	0.20
Morgue [*n* (%)]	32 (0.1)	0	0.17

^a^*χ*^2^ test. ^b^Special care: intensive care unit, coronary care unit or stroke unit. ^c^LWBS: patients left the ED without being seen by a physician.

## Discussion

Our results showed that unscheduled within-week return accounted for 5% (2,492/49,391) of our ED visits, implying an unscheduled return rate of over 200 visits a month.

Despite the Dutch health care system with universal access to primary care, our within-week unscheduled return rate (5%) was higher than in another study using a cut-off point of a week, in which 3.8% unscheduled return [[25]] was observed. One plausible explanation of our high unscheduled return rate may be that patients not always realize that they have access to a GP 24 h a day. Furthermore, patients with chronic conditions may present to the ED despite the 24-h access to the GP.

Comparison of return visit rates among studies is complicated by the different time frames used. Some studies use 72-h return visits [[2],[7],[9]–[11],[14],[16],[21]] while others have used a 30-day delay between the two visits [[26],[27]]. Applying the 72-h time frame in our results, our percentage of unscheduled return visits (2.7%) compares well with published 72-h return rates, ranging from 2.2% to 5.5% [[2],[7],[9]–[11],[14],[16],[21]]. However, our sub-analysis showed that a 72-h cut-off point would have excluded 47% of the unscheduled return visits, while risk factors were the same as those associated with unscheduled return visits within 1 week.

Some patients with an unscheduled return visit returned more than once during the week after their initial visit. They may have become ‘frequent flyers’: patients with high ED utilization, sometimes defined as patients visiting the ED seven or more times per year [[28]]. We did not follow up on our patients with unscheduled return visits, so we cannot present actual numbers on who became a frequent flyer in the 12 months after the initial visit. Frequent ED utilization, in particular by the homeless or substance abusers, seems less a problem in our ED [[29]] than outlined in the international literature [[30]].

When interpreting our medical complaint categorization as proxy measure for diagnosis, our results support the finding in a previous study [[18]] that a digestive diagnosis is a risk factor for unscheduled return. Return visits related to ‘abdominal pain’ might be explained by the difficulty of diagnosing abdominal disorders, which has a wide range of possible causes [[10]]. Emergency physicians should be particularly cautious when a patient present with a ‘high risk for return’ diagnosis, such as abdominal pain, and consider a follow-up appointment.

When using the medical complaint ‘rashes’ as proxy for dermatologic condition, our study contradicts the results in the study of Pham et al. [[11]] as ‘rashes’ was no risk factor for unscheduled return in our study. Our physicians often refer patients with rashes to the patients' GP. When these patients suffer persisting problems, they will probably return to their GP instead of to the ED.

Patients presenting with ‘chest pain’ or ‘feeling unwell’ were less likely to return unscheduled. These complaints often indicate cardiac problems. Probably these patients are either admitted at their initial visit or receive an appointment for the outpatient clinic. Parents with a ‘sick baby’ were also less likely to return. These parents are advised to go to the children's hospital in case of ongoing complaints.

In our study, over 4% of the patients lacked health insurance. Lacking health insurance was not a risk factor for unscheduled return, contradicting previous findings [[11]]. Our hospital is a regional centre for treatment of people living illegally in the Netherlands. Appointments at the outpatient clinics are arranged for anyone who needs further medical assessment after an ED visit, regardless of insurance status. Therefore, unscheduled return visits are prevented for insured and uninsured patients alike.

In previous research, conflicting findings regarding the association between triage level and unscheduled return are reported. Two studies concerning a paediatric population found that children with a high triage level were more likely to return unscheduled [[14],[16]], while in a study concerning a mixed population, returning patients had low triage levels [[11]]. In our study, patients with urgent triage levels (at their initial visit) were more likely to incur an unscheduled return visit. Possibly, patients with low triage levels were advised to return to their GP in case of persisting complaints.

Urgent triage levels may reflect a sicker patient in need for continued medical care. The longer LOS of our returning patients as compared with the LOS of patients who did not return may also indicate a sicker patient. However, our post-ED disposition data showed no sign that returning patients were more seriously ill: returning patients had similar hospital admission rates as the patients who did not return. Future studies should examine outcomes of these patients in more detail.

The percentage of illness-related reasons for unscheduled return in our study (49%) compares well with the 48% to 81% in other studies [[3],[7],[9]]. Ten percent of our unscheduled return visits were due to physician-related factors, as compared to 3% to 8% in other studies [[7],[9]]. Patient-related reasons accounted for 41% of the unscheduled return visits in our study, as compared to 11% to 53% in other studies [[6],[7],[9]]. Most patient-related returns involved patients ‘worrying about health’, indicating suitability for assessment and reassurance by the GP.

### Limitations and strengths

This study conveys the experience of a single institution and may have limited generalizability because of the social and cultural characteristics of our population and differences in health care delivery in our country. Our findings should be validated in other EDs.

Second, we used routinely collected data. This had the advantage of examining data of large numbers of patients. The disadvantage was that we were not able to account for socio-economic factors that are known to influence the probability of ED return visits, such as marital status, socio-economic status (SES), alcohol consumption and homelessness [[11],[18],[31]]. The weak discriminative capacity of our identified predictors for unscheduled return indicates that a future prospective study is needed to include these additional risk factors. However, such a study design should take into account the reliability issues associated with measuring SES and alcohol consumption in ED patients.

The categorization of the reasons of unscheduled return based on retrospective patient documentation was a limitation of our study, which we tried to limit by using explicit criteria for the categories based on previous research [[6],[9],[23]].

Another limitation is that not only patients who ‘lack health insurance’ or ‘lack a GP’ are registered as such. When it is unclear whether the patient has a health insurance and/or when the patient does not know the name of his/her GP, the patients are also registered as ‘lacking health insurance’ and/or ‘lacking a GP’. Therefore, patients might have been wrongly classified to the ‘lack health insurance’ or ‘lack GP’ group, thereby diluting a possible association between health insurance/GP-status and unscheduled return.

The strengths of this study include its complete data collection. The 11 patient records that were unavailable concerned only one patient, so selection bias was negligible. However, patients may have visited other hospital EDs after their visit to the study setting which may have led to some cases not identified.

## Conclusions

Unscheduled within-week return accounted for 5% of the ED visits. Most associated factors (an urgent triage level, arriving during the night, suffering from a wound or local infection, or a urinary problem) differ from previous studies, except for abdominal complaints, which was found to be a risk factor in many other studies. The reasons for ED return were comparable with studies from other countries: most often illness-related, then patient-related and least often physician-related reasons (e.g. ongoing complaints because a foreign body left behind in a wound or lack of a prescription of painkillers) prompted the patient back to the ED. Short-term follow-up at the outpatient clinic or GP for patients with urgent triage levels and suffering from wounds or local infections, abdominal pain or urinary problem might prevent unscheduled return.

## Abbreviations

AUC ROC: area under the receiver operating curve

CI: confidence interval

ED: emergency department

GP: general practitioner

LOS: length of stay

LWBS: left the emergency department without being seen by a physician

OR: odds ratio

SES: socio-economic status

## Competing interests

The authors declare that they have no competing interests.

## Authors' contributions

MCL had full access to all of the data in the study and takes full responsibility for the integrity of the data and the accuracy of the data analysis. MCL, RL, NL and ERJTD conceived and designed the study. MCL, NL and ERJTD acquired the data. MCL, RL, NL and CL analysed and interpreted the data. MCL and RL drafted the manuscript. RL, RH, CL, SJR and JCG critically revised the manuscript for important intellectual content. All authors read and approved the final manuscript.
